# The Trajectory of Alterations in Immune-Cell Counts in Severe-Trauma Patients Is Related to the Later Occurrence of Sepsis and Mortality: Retrospective Study of 917 Cases

**DOI:** 10.3389/fimmu.2020.603353

**Published:** 2021-01-08

**Authors:** Xijie Dong, Chuntao Wang, Xinghua Liu, Xiangjun Bai, Zhanfei Li

**Affiliations:** Trauma Center, Department of Emergency and Traumatic Surgery, Tongji Hospital of Tongji Medical College, Huazhong University of Science and Technology, Wuhan, China

**Keywords:** trauma, neutrophil, lymphocyte, monocyte, sepsis, mortality

## Abstract

**Background:**

Severe trauma is believed to disrupt the homeostasis of the immune system, and lead to dramatic changes in the circulating immune-cell count (ICC). The latter fluctuates widely over time. Knowledge about the relationship between these dramatic changes and dynamic fluctuations and the late prognosis of trauma patients is sparse. We investigated the relationship between the trajectory of alterations in the circulating ICC within 7 days in severe-trauma patients and subsequent sepsis and mortality.

**Methods:**

A retrospective analysis of 917 patients with an Injury Severity Score ≥16 was undertaken. The absolute neutrophil, lymphocyte, and monocyte counts (ANC, ALC, and AMC, respectively) on days 1, 3, and 7 (D1, D3, and D7, respectively) after trauma, and whether sepsis or death occurred within 60 days, were recorded. As the disordered circulating ICCs fluctuated widely, their time-varying slopes (D3/D1 and D7/D3) were calculated. Patients were divided into “sepsis” and “non-sepsis” groups, as well as “alive” and “death” groups. Comparative studies were conducted between every two groups. Univariate and multivariate logistic regression analyses were used to identify variables related to the risk of sepsis and mortality. Receiver operating characteristic curves were plotted to assess the predictive value of various risk factors.

**Results:**

More severe trauma caused more pronounced increases in the ANC and slower recovery of the ALC within 7 days. The ALC (D3), ANC (D7), ALC (D3/D1), and ANC (D7/D3) were independent risk factors for sepsis. The ALC (D3), ALC (D7), AMC (D7), and ALC (D3/D1) were independent risk factors for mortality. A combination of the ALC (D3) and ALC (D3/D1) exerted a good predictive value for sepsis and death.

**Conclusions:**

The trajectory of alterations in the circulating ICC in the early stage after trauma is related to subsequent sepsis and mortality.

## Introduction

Trauma carries a high morbidity and mortality. More than five million people worldwide die of trauma each year (1). It is also the primary cause of death in individuals under 40 years of age (2). The major cause of early death at the trauma scene or after hospital admission is severe brain damage or massive bleeding due to injury to the heart or large blood vessels. With advancement of resuscitation strategies and supportive care, the early death of many inpatients has been reduced considerably. However, for such early survivors, delayed death due to sepsis and multiple-organ dysfunction syndrome (MODS) is common (3–6).

Extensive injury to tissue and ischemia-induced release of damage-associated molecular patterns after severe trauma leads to a robust inflammatory response, and an important anti-inflammatory response occurs almost simultaneously (4, 7–10). These early events are, in general, believed to: (i) disrupt homeostasis of the immune system rapidly; (ii) affect the innate and adaptive arms of the immune system; (iii) lead to significant changes in the circulating number of immune cells (which are important causes of nosocomial infection, sepsis and MODS in the later stage) (4, 11–16).

Optimal management of trauma requires a clear understanding of the dramatic changes in the post-traumatic immune system and an accurate prediction of the possible poor prognosis of trauma patients to reduce medical costs (6). There is a certain understanding of the changes in immune-cell counts (ICCs) after severe trauma, such as an increase in the absolute neutrophil count (ANC) and decrease in the absolute lymphocyte count (ALC) (1). However, the relationship between the dramatic changes in the circulating ICC caused by severe trauma and the delayed onset of sepsis and death in those early survivors has yet to be elucidated. Moreover, ICC fluctuations are widespread and whether these fluctuating trends are associated with delayed sepsis and death is not known.

We investigated the changes of the ANC, ALC, and absolute monocyte count (AMC) within 7 days after severe trauma (defined as an Injury Severity Score [ISS] ≥16). We also studied the relationship between these changes and the subsequent prevalence of sepsis and death within 60 days. The disordered ICC can fluctuate widely, so focusing only on the ICC at a specific time point (the outcome of the change) may not be sufficient. The trend of increasing or decreasing of the ICC over time (the process of the change) should also be explored. Therefore, we not only investigated the relationship between the ICC and prognosis at three time points within 7 days, but also investigated the relationship between the fluctuation trend of the ICC and the prognosis.

## Methods

### Ethical Approval of the Study Protocol

This retrospective study was conducted at Tongji Hospital (Wuhan, China) from 1 January 2014 to 31 August 2020. The study was in accordance with the ethical standards set forth in the Helsinki Declaration 1964 and its later amendments. The study protocol was approved by the Medical Ethics Committee of Tongji Hospital. Informed consent was waived because this study was retrospective.

### Study Cohort

Patients admitted within 24 h after trauma with an ISS ≥16 were enrolled. The exclusion criteria were patients: (i) aged <18 years; (ii) with autoimmune diseases; (iii) with neoplastic or proliferative hematologic diseases; (iv) with severe systemic inflammation caused by other diseases (e.g., liver cirrhosis, myocardial infarction); (v) with chronic diseases necessitating immunomodulation therapy. Besides, patients who died within 7 days of study commencement were withdrawn from our study. This strategy was taken because our aim was to investigate the relationship between trauma-induced changes in the ICC at an early stage (within 7 days) and adverse outcomes that occur later (from day-7 (D7) to D60) in early survivors.

### Data Collection

Demographic data, Acute Physiology and Chronic Health Evaluation (APACHE) II score, ISS, Glasgow Coma Scale (GCS) score, the mechanism of injury, number of days of mechanical ventilation, comorbidities, whether sepsis (17) or death occurred within 60 days, and the ANC, ALC and AMC on D1, D3 and D7 were obtained from the patent-information system of Tongji Hospital.

### Statistical Analyses

Continuous and categorical variables are the mean (SD), median [interquartile range (IQR)], or number (proportion). A comparison of three correlated samples was undertaken using Friedman’s rank test (for data with a non-normal distribution) followed by the Wilcoxon signed-rank test. A comparison of two independent samples was undertaken using the Wilcoxon rank-sum test (for data with a non-normal distribution), Mann–Whitney *U*-test (for ordered categorical data), chi-square tests (for unordered categorical data) or Fisher’s exact probability test (for unordered categorical data). Univariate and multivariate logistic regression analyses were used to identify variables related to the risk of sepsis and mortality. A receiver operating characteristic (ROC) curve was plotted to calculate the area under the ROC curve (AUC). The binary logistic regression model was used for the combined analysis of two indicators. Statistical analyses and graphics were developed using SPSS v26.0 (IBM, Armonk, NY, USA), MedCalc v19.0.7 (MedCalc, Ostend, Belgium) and GraphPad Prism v8.3.0 (GraphPad, San Diego, CA, USA). *p* < 0.05 (two-sided) was considered significant.

## Results

### Cohort Characteristics

A total of 917 patients with a median (IQR) ISS of 24.0 (range, 19.0, 29.0) were screened for inclusion. **Table 1** shows the characteristics of the entire patient cohort at baseline. The prevalence of sepsis and mortality between D7 and D60 was 12.5% and 7.1%, respectively.

**Table 1:** Overall characteristics of patients suffering from severe trauma.

Variable	Patients (n = 917)
Age, mean (SD), year	46.8 (13.1)
Male, n (%)	657 (71.6)
APACHE II score, median (IQR)	11.0 (7.0, 16.0)
ISS, median (IQR)	24.0 (19.0, 29.0)
GCS score, n (%)	
3–8	151 (16.5)
9–12	145 (15.8)
13–15	621 (67.7)
Mechanism of injury, n (%)	
Fall	430 (46.9)
Road-traffic accident	415 (45.3)
Assault	30 (3.3)
Other/unknown	42 (4.6)
Duration of mechanical ventilation, days, median (IQR)	0 (0, 4.0)
Chronic medical illness, n (%)	
Hypertension	96 (10.5)
Diabetes mellitus	73 (8.0)
Coronary artery disease	45 (4.9)
Cerebrovascular disease	38 (4.1)
COPD	38 (4.1)
Sepsis, n (%)	115 (12.5)
Mortality, n (%)	65 (7.1)

APACHE II, Acute Physiology and Chronic Health Evaluation II; ISS, Injury Severity Score; GCS, Glasgow Coma Scale; COPD, chronic obstructive pulmonary diseases.

### More Severe Trauma Leads to Worse Alterations in the ICC

**Figure 1:**
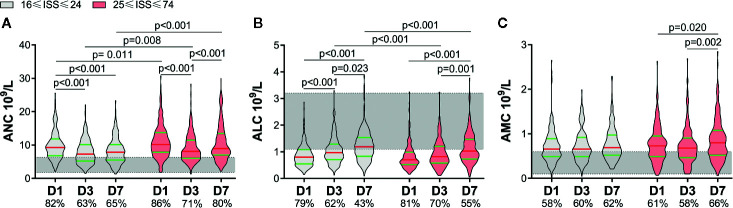
Alterations of circulating immune-cell counts in severe-trauma patients within 7 days. The proportion of patients with a value above the upper limit of the normal range (shaded area) of the absolute neutrophil count (ANC) and absolute monocyte count (AMC) or below the lower limit of the normal range of the absolute lymphocyte count (ALC) are shown on the x-axis. An increase in the ANC and AMC **(A, C)**, and decrease in the ALC **(B)** were common in severe-trauma patients. ISS, Injury Severity Score; D1, day-1; D3, day-3; D7, day-7.

According to the ISS, patients were divided into two groups: 16≤ ISS ≤24 and 25≤ ISS ≤75. The latter had a higher APACHE II score, lower GCS score, and longer duration of mechanical ventilation [**Supplemental Digital Content (SDC): eTable 1**]. An increase in the ANC and AMC, and decrease in the ALC, were common in severe-trauma patients (**Figures 1A–C**). Patients with a higher ISS had a higher ANC within 7 days. Although the ANC of both groups showed a decline on D3, it rebounded in patients with a higher ISS on D7 (**Figure 1A**). There was no significant difference in the ALC between the two groups on D1. Then, the ALC of the lower-ISS group increased on D3, whereas the ALC of the higher-ISS group increased on D7. Also, the ALC of the latter was less than that of the former on D3 and D7 (**Figure 1B**). Besides, the AMC increased in the higher-ISS group on D7, but there was no significant difference between the two groups (**Figure 1C**).

### Patients With Subsequent Sepsis Have Worse Early Alterations in the ICC

**Figure 2:**
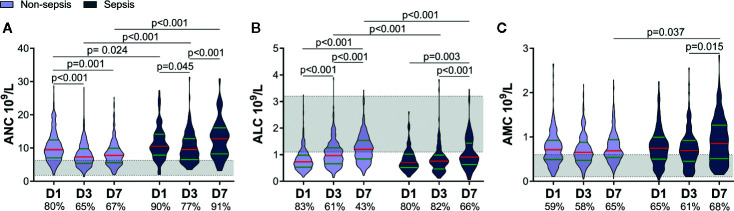
Patients who later developed sepsis had worse early alterations of circulating immune-cell counts. The proportions of patients with a value above the upper limit of the normal range (shaded area) of the absolute neutrophil count (ANC) and absolute monocyte count (AMC) or below the lower limit of the normal range of the absolute lymphocyte count (ALC) are shown on the x-axis **(A–C)**. D1, day-1; D3, day-3; D7, day-7.

According to whether sepsis occurred within 60 days, we divided patients into two groups: non-sepsis and sepsis. A total of 115 patients developed sepsis, with the lung being the most common site of infection, and Gram-negative bacteria being the most common pathogen. The sepsis group had a higher ISS and APACHE II score, lower GCS score, and longer duration of mechanical ventilation (SDC: **eTable 1**). Differences in the ANC and ALC between the non-sepsis group and sepsis group within 7 days were similar to those between the lower-ISS group and higher-ISS group (**Figures 1A, B**, **2A, B**). Sepsis patients had a higher ANC within 7 days. Although the ANC of both groups showed a decrease on D3, it rebounded in sepsis patients on D7 (**Figure 2A**). There was no significant difference in the ALC between the two groups on D1. Then, the ALC of non-sepsis patients increased on D3, whereas the ALC of sepsis patients increased on D7. The ALC of the latter was less than that of the former on D3 and D7 (**Figure 2B**). Besides, the AMC of the sepsis group increased on D7, and was higher than that in the non-sepsis group (**Figure 2C**).

### Patients Who Die Subsequently Have Worse Early Alterations in the ICC

**Figure 3:**
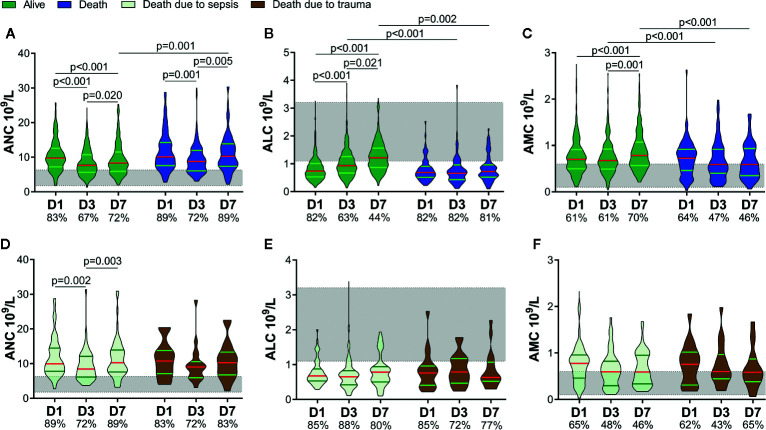
Circulating immune-cell counts of living and dead patients. Circulating immune-cell counts showed worse early alterations in non-survivors. They did not differ between non-survivors who died of sepsis and those who died of trauma. The proportions of patients with an absolute neutrophil count (ANC) and absolute monocyte count (AMC) above the upper limit of the normal range (shaded area) are shown on the x-axis **(A, C**, **D, F)**. The proportions of patients with an absolute lymphocyte count (ALC) below the lower limit of the normal range (shaded area) are shown on the x-axis **(B, E)**. D1, day-1; D3, day-3; D7, day-7.

According to whether death occurred within 60 days, we divided patients into two groups: alive and death. Sixty-five patients with a higher ISS and APACHE II score, lower GCS score, and longer duration of mechanical ventilation were enrolled in the death group (SDC: **eTable 1**). The ANC of both groups decreased on D3 and rebounded on D7, but the ANC of the death group increased to the same level as that on D1 on D7. There was a difference in the ANC between the two groups on D7 (**Figure 3A**). There was no difference in the ALC between the two groups on D1, but it escalated in the alive group, and no significant change in the death group within 7 days was noted. As a result, the ALC differed between the two groups on D3 and D7 (**Figure 3B**). Besides, the AMC increased on D7 in the alive group and was higher than that in the death group on D3 and D7 (**Figure 3C**).

We further divided the non-survivors into two groups depending on the cause of death: sepsis and trauma. Thirty-seven patients died of sepsis and 28 patients died of trauma. There were no significant differences in age, sex, ISS, APACHE II score, or GCS score between the two groups, or indeed the ANC, ALC, or AMC (SDC: **eTable 1** and **Figures 3D–F**). These results implied that, regardless of whether patients died eventually of sepsis or trauma, they might suffer a similar degree and potentially fatal insult to the immune system in the early stage.

### Subsequent Sepsis and Death Are Related to Time-Varying Slopes of the ICC

**Figure 4:**
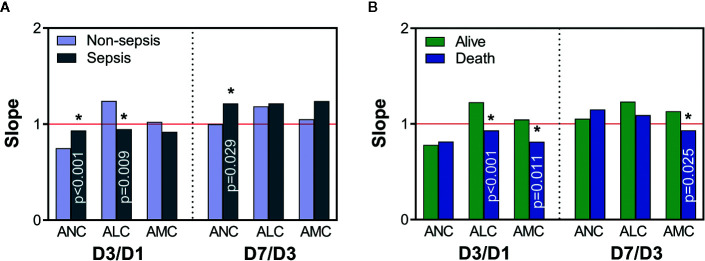
The time-varying slope of immune-cell counts. Comparisons of slopes between non-sepsis and sepsis groups **(A)**, and alive and death groups **(B)**. A value of slope >1 (red line) indicates an upward trend in immune-cell counts, and a value <1 indicates a downward trend; *Represents a significant difference between non-sepsis and sepsis groups or alive and death groups. ANC, absolute neutrophil count; ALC, absolute lymphocyte count; AMC, absolute monocyte count; D1, day-1; D3, day-3; D7, day-7.

Through the analyses mentioned above, we found that sepsis and death may be related not only to the ICC at a certain time point, but also to its upward and downward trends. We wished to study further the relationship between the fluctuations of the ICC over time and later occurrence of sepsis and death. Hence, we investigated the time-varying slopes of the ICC within 7 days, which more clearly reflected the early-change trajectory of immune cells. A slope >1 shows an upward trend, and a larger value indicates a greater increase; a slope <1 shows a downward trend, and a smaller value indicates a more pronounced decrease. The ANC (D3/D1), ALC (D3/D1), and ANC (D7/D3) differed between the non-sepsis and sepsis groups (**Figure 4A**). This finding suggested that the decline in ANC (slope <1) and increase in the ALC (slope >1) were greater in non-sepsis patients on D1 to D3, and that the rise in the ANC (slope >1) was more pronounced in sepsis patients on D3 to D7. The ALC (D3/D1), AMC (D3/D1), and AMC (D7/D3) were >1 in survivors and <1 in non-survivors (**Figure 4B**). Hence, in survivors, the ALC increased on D1 to D3 and AMC increased on D1 to D7, whereas the opposite was true in non-survivors.

### Analyses of Risk Factors and Comparison of Predictive Value

We undertook risk-factor analyses to further determine the relationship between the ICC and sepsis and mortality (**Table 2**). Analyses of the ICC at fixed time points and the slopes of each two time points were evaluated separately to prevent collinearity. After adjustment for confounding factors, we discovered that the ISS, APACHE II score, ANC (D7), ALC (D3), ANC (D7/D3), and ALC (D3/D1) were associated significantly with sepsis; the ISS, APACHE II score, GCS, ALC (D3), ALC (D7), AMC (D7), and ALC (D3/D1) were associated significantly with mortality.

**Table 2:** Univariate and multivariate logistic regression analysis.

Variable	Sepsis				Mortality			
	Univariate analysis		Multivariate analysis		Univariate analysis		Multivariate analysis	
	OR (95% CI)	*p*	OR (95% CI)	*p*	OR (95% CI)	*p*	OR (95% CI)	*p*
Age	1.02 (1.00, 1.04)	0.024	1.00 (0.99, 1.02)	NS	1.03 (1.02, 1.07)	0.001	0.99 (0.97, 1.03)	NS
ISS	1.11 (1.08, 1.14)	<0.001	1.05 (1.02, 109)	0.015	1.08 (1.06, 1.11)	<0.001	1.05 (1.02, 1.08)	0.012
APACHE II	1.18 (1.13, 1.23)	<0.001	1.18 (1.11, 1.24)	0.001	1.24 (1.20, 1.30)	<0.001	1.28 (1.18, 1.37)	<0.001
GCS score	0.82 (0.77, 0.88)	0.002	1.07 (0.98, 1.16)	NS	0.81 (0.75, 0.88)	0.004	1.09 (1.02, 1.17)	0.041
Immune cell counts								
ANC (D1)	1.04 (1.01, 1.09)	0.016	0.99 (0.91, 1.05)	NS	1.04 (1.00, 1.11)	NS		
ANC (D3)	1.18 (1.09, 1.26)	<0.001	1.09 (0.99, 1.20)	NS	1.06 (1.01, 1.12)	0.034	1.05 (0.96, 1.17)	NS
ANC (D7)	1.23 (1.16, 1.31)	<0.001	1.16 (1.08, 1.26)	<0.001	1.07 (1.03, 1.12)	0.003	1.07 (0.98, 1.16)	NS
ALC (D1)	0.95 (0.58, 1.59)	NS			1.08 (0.64, 1.92)	NS		
ALC (D3)	0.55 (0.32, 0.84)	0.034	0.46 (0.22, 0.90)	0.026	0.33 (0.17, 0.65)	0.002	0.38 (0.18, 0.82)	0.018
ALC (D7)	0.54 (0.37, 0.83)	0.005	0.60 (0.34, 1.08)	NS	0.15 (0.08, 0.31)	<0.001	0.28 (0.11, 0.68)	0.007
AMC (D1)	1.37 (0.79, 2.35)	NS			1.36 (0.73, 2.54)	NS		
AMC (D3)	1.29 (0.74, 2.26)	NS			0.47 (0.22, 1.03)	NS	1.32 (0.42, 4.13)	NS
AMC (D7)	2.24 (1.31, 3.54)	0.008	1.74 (0.76, 3.96)	NS	0.25 (0.12, 0.52)	<0.001	0.22 (0.06, 0.72)	0.026
Slope								
ANC (D3/D1)	1.51 (1.04, 2.19)	0.024	1.49 (0.93, 2.37)	NS	1.20 (0.79, 1.81)	NS		
ANC (D7/D3)	1.70 (1.24, 2.35)	0.001	1.63 (1.15, 2.34)	0.007	1.13 (0.80, 1.58)	NS		
ALC (D3/D1)	0.71 (0.53, 0.95)	0.012	0.71 (0.56, 0.96)	0.048	0.69 (0.48, 0.91)	0.013	0.71 (0.50, 0.95)	0.040
ALC (D7/D3)	1.35 (1.04, 1.67)	0.041	1.16 (0.86, 1.55)	NS	0.86 (0.61, 1.18)	NS		
AMC (D3/D1)	1.05 (0.94, 1.17)	NS			1.05 (0.96, 1.15)	NS		
AMC (D7/D3)	1.25 (1.03, 1.4)	0.033	1.10 (0.91, 1.34)	NS	0.93 (0.71, 1.17)	NS		

Table shows the results of variables related to sepsis and mortality determined by univariate and multivariate logistic regression analysis. To avoid collinearity, immune-cell counts and their slopes were evaluated separately. OR, odds ratio; CI, confidence interval; ISS, Injury Severity Score; APACHE II, Acute Physiology and Chronic Health Evaluation II; GCS, Glasgow Coma Scale; ANC, absolute neutrophil count; ALC, absolute lymphocyte count; AMC, absolute monocyte count; D1, day-1; D3, day-3; D7, day-7; NS, not significant.

**Figure 5:**
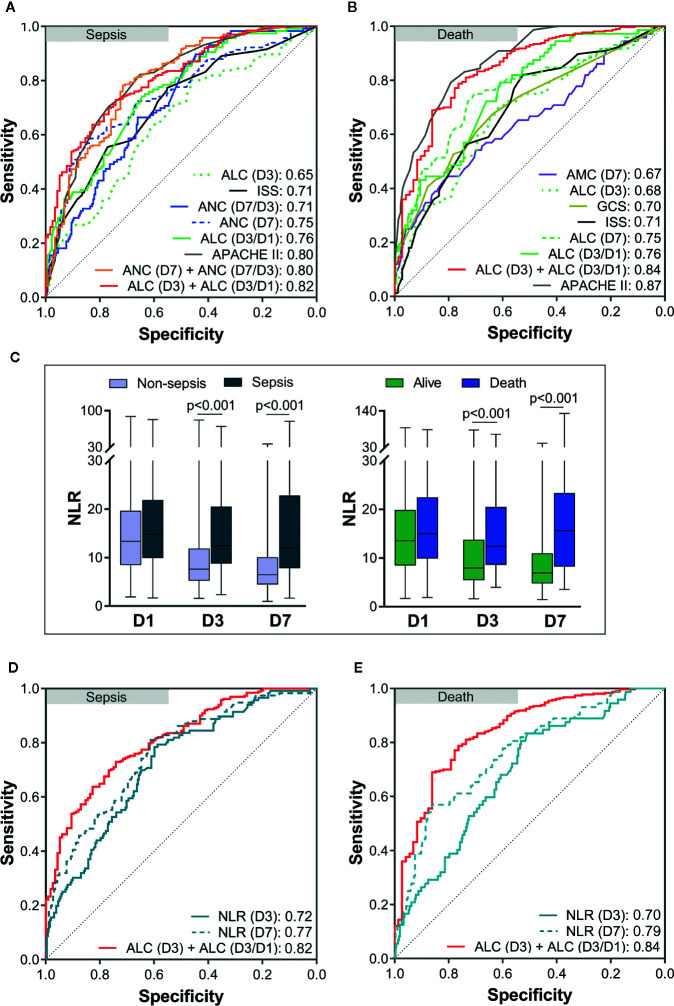
Analyses of receiver operating characteristic (ROC) curves to compare the predictive values of risk factors. The areas under the ROC curves (AUC) are presented in the figure. The predictive value of risk factors and the combinations of some risk factors for sepsis **(A)** and death **(B)**. Comparison of the neutrophil:lymphocyte ratio (NLR) between each two groups **(C)**. The predictive value of the NLR (D3) and NLR (D7) for sepsis **(D)** and death **(E)** was lower than that of a combination of the ALC (D3) and ALC (D3/D1). APACHE II, Acute Physiology and Chronic Health Evaluation II; ISS, Injury Severity Score; GCS, Glasgow coma scale; ANC, absolute neutrophil count; ALC, absolute lymphocyte count; AMC, absolute monocyte count; D1, day-1; D3, day-3; D7, day-7.

Then, ROC curves were used to assess the predictive value of risk factors and the combinations of some risk factors for sepsis and death in severe-trauma patients (**Figures 5A, B**). The combination of the ICC and its time-varying slope provided better predictive value than either parameter alone. A combination of the ALC (D3) and ALC (D3/D1) had the best predictive value (AUC: 0.82) for sepsis (**Figure 5A**). For mortality, although the predicted value of the combination of ALC (D3) and ALC (D3/D1) was inferior to that of the APACHE II score (AUC: 0.84 VS 0.87), it was better than other predictors (**Figure 5B**). Besides, the neutrophil:lymphocyte ratio (NLR) has been shown to be a simple and reliable marker for assessing systemic inflammation and prognosis in non-traumatic and traumatic patients (1, 18–22). Therefore, we also validated the predicted value of the NLR (D3) and NLR (D7) given their differences between each two groups (**Figure 5C**). We found that the AUC of a combination of ALC (D3) and ALC (D3/D1) was larger than that of the NLR (D3) and NLR (D7) in predicting the risk of sepsis and death (**Figures 5D, E**).

## Discussion

We revealed the trajectory of alterations in the ANC, ALC, and AMC within 7 days after severe trauma, and unveiled the relationship between these alterations and the later occurrence of sepsis and death. These three types of immune cells in peripheral blood cover most innate and adaptive immunocytes, and their absolute counts can be detected readily. They have more practical value compared with other indicators that are not easy to measure. We showed that more severe trauma may lead to more severe interference in the immune system, and that worse early alterations in the ICC are associated with the later occurrence of sepsis and death.

Neutrophils can carry out strong phagocytosis of pathogens and are on the “frontline” of the body’s defense against pathogenic microorganisms (23). However, they can also damage blood vessels and surrounding tissues by releasing lysosomal enzymes, and an uncontrolled innate immune system response can make sepsis and organ failure more likely to occur (1, 5, 24, 25). In our study, patients with more severe trauma had a higher ANC within 7 days, which indicated that more severe trauma may lead to more severe interference of the innate immune system. Patients with subsequent sepsis also had a higher ANC within 7 days. Also, the ANC of patients with subsequent sepsis showed a smaller decrease on D1 to D3, and rebounded significantly on D3 to D7 according to the results of the slope. Moreover, the ANC (D7) and ANC (D7/D3) were shown to be independent risk factors of sepsis. These results imply that secondary sepsis may be associated with a higher ANC due to more severe trauma. Besides, the ANC (D7) in non-survivors was higher than that in survivors, but multivariate analyses showed that it was not an independent risk factor for death. Therefore, we cannot assume that a higher ANC will increase the risk of death.

Lymphocytes have a central role in the anti-infective immune response because they can interact with cells of the innate immune system and other cells associated with adaptive immunity (26). They are not “passive bystanders”; they play a key part in appropriate regulation of the inflammatory response (26, 27). Post-traumatic lymphopenia was common in the patients in our study. Our results are similar to findings in critically ill patients, such as those suffering from trauma and sepsis. The extent of and delayed recovery from lymphopenia have been reported to be related to nosocomial infection, sepsis, and death (26, 28–33). Interestingly, we found that more severe trauma did not lead initially to more severe depletion of lymphocytes, but it caused a lower ALC on D3 and D7. Patients who suffered sepsis subsequently and who died also had a lower ALC on D3 and D7. This finding may have been due to the slower recovery of the ALC between D1 to D3 because there was no difference in the ALC (D7/D3) between non-sepsis and sepsis patients, and survivors and non-survivors, according to the results of the slope. Multivariate analyses showed that the ALC (D3) and ALC (D3/D1) were independent risk factors for sepsis, and that the ALC (D3), ALC (D7), and ALC (D3/D1) were independent risk factors for mortality. Taken together, these results imply that more severe trauma may cause the ALC to recover more slowly within 3 days so that the ALC is lower on D3 and D7, which may increase the risk of sepsis and death. In addition, ROC analyses showed that a combination of the ALC (D3) and ALC (D3/D1) was the best predictor of sepsis, and was the next-best predictor of death. Hence, a combination of the ALC (D3) and ALC (D3/D1) could be used as an important predictive tool for a poor prognosis after trauma.

Monocytes are the precursors of macrophages. They can phagocytize pathogens, mediate and promote the inflammatory response, process and present antigens, and participate in regulating the adaptive immune response (34). Monocyte dysfunctions (e.g., reduced expression of human leukocyte antigen–DR isotype and a reduced ability to release inflammatory factors) have been investigated widely in severe trauma and sepsis (4, 35–40). However, there are few reports of changes in their counts. In our study, the AMC increased on D7 and was higher in patients who had sepsis subsequently. The overall rise of the AMC after trauma may have been due to endogenous stimulation (damaged tissue), and this increase on D7 may have been caused by new exogenous stimulation (microbial infection). The AMC was higher in survivors than in non-survivors on D3 and D7. This result is in accordance with the findings of two recent studies which reported that survivors have a significantly higher AMC than non-survivors in critically ill patients (41, 42). Furthermore, we found the changing trend of the AMC in survivors and non-survivors was contrary, increasing in the alive group (slope >1) and falling in the death group (slope <1) within 7 days. Besides, multivariate analyses showed that the AMC (D7) was an independent risk factor for mortality. Collectively, considering the important role of monocytes and the fact that some monocytes have functional impairments, ensuring more cell counts may mitigate the adverse consequences caused by the dysfunction of some cells.

Our study had three main limitations. First, this was a single-center retrospective study. The nature of our study may have led to an unintentional bias in patient selection. Second, blood transfusion can affect the function of the immune system (43, 44), but the interference of blood transfusion was not excluded in our study. Third, the immune-cell function was not investigated in this retrospective study, but it is also an important part of post-traumatic immune-cell dysfunction. Our findings need to be verified by large, prospective multi-center studies of rigorous design.

## Conclusions

The trajectory of early changes in the ICC was associated with subsequent sepsis and death. More severe trauma caused more pronounced increases in the ANC and slower recovery of the ALC in the early stage after trauma. Patients who developed sepsis subsequently were characterized by a higher initial ANC that declined slowly and rebounded on D7, and a similar initial level of the ALC but a slower recovery in D1 to D3. Patients who died subsequently were also characterized by an ALC that was at the same level initially but recovered more slowly in D1 to D3, and with an AMC of a similar level initially but a continuous downward trend within 7 days. Furthermore, a combination of the ALC (D3) and ALC (D3/D1) exerted a good predictive value for the risk of sepsis and mortality in severe-trauma patients, which can help to identify the delayed poor prognosis early and aid early intervention. Based on these findings, as well as the harmfulness of an excessive ANC (1, 24, 25), the protective value of a sufficient ALC (27–29, 45), and reduction of the functional AMC (4, 37, 38), we speculate that immune interventions such as clearing an excessive dysfunctional ANC, recovering the ALC as soon as possible, and supplementing the AMC with normal function may help to improve the prognosis and may be worthy of further study.

## Data Availability Statement

The raw data supporting the conclusions of this article will be made available by the authors, without undue reservation.

## Ethics Statement

The studies involving human participants were reviewed and approved by Medical Ethics Committee of Tongji Hospital of Tongji Medical College of Huazhong University of Science and Technology. Written informed consent for participation was not required for this study in accordance with the national legislation and the institutional requirements.

## Author Contributions

Study conception and design: XD, ZL, and XB. Acquisition of data: XD, XL, and CW. Analysis and interpretation of data: XD, CW, ZL, and XB. Drafting of the article: XD, XL, and CW. Critical revision: ZL and XB. All authors contributed to the article and approved the submitted version.

## Funding

This work was supported by National Natural Science Foundation of China (No. 81571891 and 81772129).

## Conflict of Interest

The authors declare that the research was conducted in the absence of any commercial or financial relationships that could be construed as a potential conflict of interest.
