# Supplementary material for: The Trajectory of Alterations in Immune-Cell Counts in Severe-Trauma Patients Is Related to the Later Occurrence of Sepsis and Mortality: Retrospective Study of 917 Cases
Source: Front Immunol. 2021 Jan 8;11:603353. doi: 10.3389/fimmu.2020.603353 (PMC7820769; doi:10.3389/fimmu.2020.603353)
Supplement: Supplementary file 1 [file Table_1.docx]

**Supplemental Digital Content**

**eTable 1. Demographic data of trauma patients grouped based on Injury Severity Score and prognosis**

| Variables | **ISS** | | |  | **Sepsis** | | |  | **Outcome** | | |  | **Cause of death** | | |
| --- | --- | --- | --- | --- | --- | --- | --- | --- | --- | --- | --- | --- | --- | --- | --- |
|  | 16-24 | 25-74 | *p* |  | No | Yes | *p* |  | Alive | Death | *p* |  | Sepsis | Trauma | *p* |
|  | (n=516) | (n=401) |  |  | (n=802) | (n=115) |  |  | (n=852) | (n=65) |  |  | (n=37) | (n=28) |  |
| Age, mean (SD), year | 47.6 (11.9) | 46.2 (13.2) | NS |  | 45.3 (11.2) | 47.9 (13.8) | NS |  | 45.0 (11.0) | 48.5 (14.3) | NS |  | 50.6 (14.7) | 47.3 (15.1) | NS |
| Male, n (%) | 365 (70.7) | 291 (72.6) | NS |  | 570 (71.1) | 87 (75.7) | NS |  | 605 (71.0) | 52 (80.0) | NS |  | 29 (78.4) | 23 (82.1) | NS |
| ISS, median (IQR) |  |  |  |  | 22.0 (19.0, 27.0) | 29.0 (24.0, 36.0) | <0.001 |  | 22.0 (19.0, 29.0) | 29.0 (24.0, 34.0) | <0.001 |  | 29.0 (24.0, 36.0) | 29.0 (24.0, 34.0) | NS |
| APACHE II, median (IQR) | 8(6.0, 13.0) | 12.0 (8.0, 19.0) | <0.001 |  | 8.0 (6.0, 13.0) | 16.0 (11.0, 23.0) | <0.001 |  | 9.0 (6.0, 14.0) | 19.0 (14.0, 26.0) | <0.001 |  | 18.5 (14.0, 24.8) | 21.5 (13.5, 27.0) | NS |
| GCS, n (%) |  |  | <0.001 |  |  |  | <0.001 |  |  |  | <0.001 |  |  |  | NS |
| 3-8 | 51 (9.9) | 100 (24.9) |  |  | 110 (13.7) | 41 (35.7) |  |  | 122 (14.3) | 29 (44.6) |  |  | 13 (35.1) | 16 (57.1) |  |
| 9-12 | 68 (13.2) | 77 (19.2) |  |  | 118 (14.7) | 27 (23.5) |  |  | 132 (15.5) | 13 (20.0) |  |  | 8 (21.6) | 5 (17.93) |  |
| 13-15 | 397 (76.9) | 224 (55.9) |  |  | 574 (71.6) | 47 (40.9) |  |  | 598 (70.2) | 23 (35.4) |  |  | 16 (43.2) | 7 (25.0) |  |
| Ventilator days, median (IQR) | 0 (0, 0) | 0 (0, 8) | <0.001 |  | 0 (0, 0) | 7 (0, 11) | <0.001 |  | 0 (0, 0) | 9.0 (5.0, 11.3) | <0.001 |  | 9.0 (6.0, 12.5) | 8.5 (5.0, 11.8) | NS |
| Co-morbidities, n (%) |  |  | NS |  |  |  | NS |  |  |  | NS |  |  |  | NS |
| Hypertension | 58 (11.2) | 38 (9.5) |  |  | 83 (10.3) | 13 (11.3) |  |  | 90 (10.6) | 6 (9.2) |  |  | 3 (8.1) | 3 (10.7) |  |
| Diabetes mellitus | 46 (8.9) | 27 (6.7) |  |  | 60 (7.5) | 13 (11.3) |  |  | 67 (7.9) | 6 (9.2) |  |  | 4 (10.8) | 2 (7.1) |  |
| Coronary artery disease | 23 (4.5) | 22 (5.5) |  |  | 38 (4.7) | 7 (6.1) |  |  | 41 (4.8) | 4 (6.2) |  |  | 3 (8.1) | 1 (3.6) |  |
| Cerebrovascular disease | 23 (4.5) | 15 (3.7) |  |  | 34 (4.2) | 4 (3.5) |  |  | 36 (4.2) | 2 (3.1) |  |  | 1 (2.7) | 1 (3.6) |  |
| COPD | 26 (5.0) | 12 (3.0) |  |  | 30 (3.7) | 8 (7.0) |  |  | 31 (3.6) | 8 (12.3) |  |  | 6 (16.2) | 2 (7.1) |  |

APACHE II score = Acute Physiology and Chronic Health Evaluation II score; ISS = Injury Severity Score; GCS = Glasgow coma scale; COPD = chronic obstructive pulmonary diseases; NS = not significant.
